# In Vitro and In Vivo Response to Low-Modulus PMMA-Based Bone Cement

**DOI:** 10.1155/2015/594284

**Published:** 2015-08-20

**Authors:** Elin Carlsson, Gemma Mestres, Kiatnida Treerattrakoon, Alejandro López, Marjam Karlsson Ott, Sune Larsson, Cecilia Persson

**Affiliations:** ^1^Division of Orthopedics, Department of Surgical Sciences, Uppsala University Hospital, Entrance 61, 751 85 Uppsala, Sweden; ^2^Division of Applied Materials Science, Department of Engineering Sciences, Ångström Laboratory, Uppsala University, P.O. Box 534, 751 21 Uppsala, Sweden

## Abstract

The high stiffness of acrylic bone cements has been hypothesized to contribute to the increased number of fractures encountered after vertebroplasty, which has led to the development of low-modulus cements. However, there is no data available on the in vivo biocompatibility of any low-modulus cement. In this study, the in vitro cytotoxicity and in vivo biocompatibility of two types of low-modulus acrylic cements, one modified with castor oil and one with linoleic acid, were evaluated using human osteoblast-like cells and a rodent model, respectively. While the in vitro cytotoxicity appeared somewhat affected by the castor oil and linoleic acid additions, no difference could be found in the in vivo response to these cements in comparison to the base, commercially available cement, in terms of histology and flow cytometry analysis of the presence of immune cells. Furthermore, the in vivo radiopacity of the cements appeared unaltered. While these results are promising, the mechanical behavior of these cements in vivo remains to be investigated.

## 1. Introduction

Poly(methyl methacrylate) (PMMA) is a synthetic thermosetting polymer that has been used as the base to produce bone cements for orthopedics since the 1960s, when Charnley first reported its use to anchor endoprostheses to bone [1, 2]. PMMA has remained popular despite the emergence of other biomaterials that bear greater resemblance to bone, such as calcium phosphate cements, due to its high strength and ductility in comparison to the ceramic cements. PMMA-based cements are currently used in a variety of applications, such as joint arthroplasty, percutaneous vertebroplasty, and kyphoplasty [3, 4].

In spite of their success in vertebroplasty—they provide pain relief and stability to the fracture site—acrylic bone cements present some issues. Their high stiffness in comparison to that of cancellous bone results in a property mismatch that has been hypothesized to be a contributing factor to adjacent vertebral fractures occurring shortly after vertebroplasty [5–8]. In fact, most commercial acrylic bone cements have an elastic modulus in the range of 1700–3700 MPa [9, 10], while the elastic modulus of vertebral trabecular bone is typically in the range of 10–750 MPa [11–13], encompassing osteoporotic to healthy bone. Therefore, cements with a lower stiffness are desired in order to potentially decrease the risk of additional fractures, and such cements have been the object of investigation by different research groups.

Unsaturated fatty acids and their glycerol esters are both natural compounds that can be used to modify the properties of bone cements. For instance, Vázquez et al. used aromatic amines as well as acrylic monomers, both derived from oleic acid, to optimize the properties of bone cements [14]. These authors reported similar compressive strengths and a reduction of 62.3% in Young's modulus when a modified liquid phase containing 2.57 wt% 4-N,N-dimethylaminobenzyl oleate was used. Lam et al. also modified their cements with strontium-substituted hydroxyapatite-nanoparticles and linoleic acid and reported similar compressive strengths accompanied by a reduction of 63.9% in Young's modulus when 20 wt% nanoparticles with 15 vol% linoleic acid were incorporated [15]. However, none of these cements are commercially available. In a previous study, we partially substituted the monomer with castor oil and found that adding up to 12% of this triglyceride decreased the compressive strength and modulus by 83% and 70%, respectively. However, the modified cements gave a reduced cell viability in a worst-case scenario [16]. Further, preliminary testing has suggested that a modification of the monomer-to-additive ratio could improve biocompatibility, but this remains to be confirmed. The authors have also showed that, using only small amounts (≤1.5 wt%) of linoleic acid, it was possible to decrease the compressive strength and Young's modulus of a commercial bone cement by 76% and 83%, respectively, with initial cytotoxicity tests showing promising results. While promising mechanical data is available for these modified cements, their in vitro and in vivo biocompatibility remain to be confirmed [17]. In fact, to the authors knowledge, no in vivo study is currently available for any low-modulus acrylic cement.

The present work hence aimed to evaluate castor oil- and linoleic acid-modified low-modulus acrylic bone cements both in vitro, using an osteoblastic-like cell model, and in vivo, using a subcutaneous rat model. Cell viability was evaluated on human osteoblast-like Saos-2 cells and the in vivo response was evaluated using histology and flow cytometry after implantation in Sprague-Dawley rats. The radiopacity of the modified cements was confirmed using in vivo microtomography.

## 2. Materials and Methods

### 2.1. Cement Preparation

OsteopalV (OP, Heraeus Medical GmbH, Hanau, Germany) radiopaque bone cement for vertebroplasty was used as the base cement. 12.3 wt% (of total cement weight) castor oil (CO, Sigma Aldrich, 259853, St Louis, MO, USA) was used, corresponding to 1.78 g CO for 10.0 g of OsteopalV powder and 2845 *μ*L monomer liquid. 1.5 wt% 9-cis,12-cis-linoleic acid (LA, ≥99%, Sigma-Aldrich, reference number W338001) was used, corresponding to 226 *μ*L LA for 10.0 g of powder and 3620 *μ*L liquid. These formulations were found to be advantageous to the in vitro biocompatibility in preliminary studies [17, 18]. Each batch of bone cement was prepared by adding the modified monomer phase to the (unaltered) powder phase in a glass mortar and mixing it by hand with a metal spatula for 1 minute. The nomenclature used in this paper indicates whether the cement contains no additive (OP) or whether it is modified with LA (OP + LA) or CO (OP + CO).

Disc-shaped cement samples (*⌀* = 6 or 13 mm, *h* = 2 mm) were molded and allowed to set for 1 h at room temperature. Cement samples to be evaluated in vivo were kept under sterile conditions and placed in separate containers of PBS (Dulbecco's phosphate buffered saline, pH 7.4, Sigma) at 37°C and allowed to set for another 24 h. All the materials were prepared under aseptic conditions.

### 2.2. In Vitro Study

The cytotoxicity of unmodified OsteopalV and of the low-modulus cements was evaluated by an indirect contact assay in which cells were cultured with cement extract (medium having been in contact with cement). Human osteoblast-like Saos-2 cells (HPACC) were used as the cell model. The cells were maintained in cell culture flasks in an incubator with a humidified atmosphere of 5% CO_2_ in air at 37°C. DME/F-12 medium (Thermo Scientific HyClone, reference number SH300023.01, Logan, UT, USA) supplemented with 1% penicillin/streptomycin (Sigma Aldrich, reference number P4333, St. Louis, Mo, USA) and 10% foetal bovine serum (Thermo Scientific HyClone, reference number SV30160.03, Logan, UT, USA) was used as culture medium. The medium was exchanged every second day. Upon confluence, cells were detached with a minimum amount of trypsin 0.25% in EDTA (Thermo Scientific HyClone, reference number SH30042.02, Logan, UT, USA) that was inactivated with supplemented medium after 10 min.

Cement extracts were prepared by immersing a cement disk (*⌀* = 13 mm, *h* = 2 mm) in 0.63 mL of complete media. The surface-to-volume ratio, which corresponded to 3 cm^2^/mL, was selected to fulfill the ISO standard ISO-10993-11 [19]. To investigate a time-dependent release of toxic by-products, the media in contact with the cement were withdrawn after 1, 6, 12, and 24 h and replaced by fresh culture medium. The extracts were sterilized by filtration using a 0.2 *μ*m pore membrane.

6500 Saos-2 cells were seeded in a 96-well plate (2 × 10^4^ cells/cm^2^) and were cultured for 24 h before starting the cytotoxicity assay. After 24 h, media were replaced by cement extract, which was added to the cells as obtained (100%), diluted 4-fold (25%) and diluted 10-fold (10%). Complete media were used as negative control (C−), media containing 0.1% Triton X-100 (Merck, reference number 1.08603.1000) were used as positive control (C+), and wells without cells were used as blank. Four replicates were included per sample.

Cells were incubated either for 1 day or 3 days, and cell viability was tested by AlamarBlue assay (Invitrogen, reference number DAL1100, Carlsbad, CA, USA). For this purpose, cells were washed once with PBS and afterwards 200 *μ*L of 5% AlamarBlue/MEM (Life Technologies, Gibco, reference number 51200, Carlsbad, CA, USA) was added to each well. After incubation in the dark for 1 h at 37°C, fluorescence was monitored on a microplate reader (Infinite M200, Tecan, Männedorf, Switzerland) at 560 nm excitation and 590 nm emission. The results were converted to cell numbers using a calibration curve.

### 2.3. In Vivo Study

#### 2.3.1. Animals and Experimental Design

The animal study was approved by the local ethical committee (Approval number C208/12). In total, 18 male Sprague-Dawley rats, weighing 400–450 g (Taconic Farms Inc., Denmark), were used. The animals were randomly distributed into three groups (3 time points, 1, 4, and 12 weeks), and all individuals received implants of all three material compositions.  Table 1 summarizes the design of the in vivo study. To keep the number of animals used at a minimum, each animal received eight implants in total. This allows for the possibility to analyze a high number of implants, while the implant sites are still not too close to each other, and more importantly the animals are basically unaffected by the procedure.

The base material (unmodified OsteopalV) was used as the control. The rats were kept in pairs in Macron 4 cages, at the animal facility at Uppsala University Hospital, with daily monitoring by the animal facility personnel. The end points were chosen based on the three contact duration categories recommended for biomaterials and medical devices: (1) limited contact (<24 h), (2) prolonged contact (>24 h and <30 days), and (3) permanent contact (>30 days) [20]. Acrylic bone cements are generally placed in categories (2) and (3), and thus the end points were chosen accordingly. At the chosen time points (1, 4, and 12 weeks) the rats were euthanized in a CO_2_ chamber.

#### 2.3.2. Surgical Procedure

The surgeries were performed under aseptic conditions. The rats were anaesthetized in an induction chamber with 5% isoflurane (Baxter, reference number KDG9623, Kista, Sweden), 0.3 L/min oxygen, and 0.7 L/min nitrous oxide for a few minutes and then transferred to an anesthesia mask (the anesthesia reduced to 1–2.5% isoflurane, 1.0 L/min oxygen, and 0.8 L/min nitrous oxide). One dose of 225 mg/kg antibiotics (Zinacef, GlaxoSmithKline AB, Sweden) was administered subcutaneously. The animals were placed on a heated pad (37°C) and the anterolateral back was shaved and disinfected with chlorhexidine (5 mg/mL; Fresenius Kabi, reference number 53 80 58, Uppsala, Sweden) and ethanol (70%). Eight cement discs (*⌀*: 6 mm; *h*: 2 mm) were placed subcutaneously, four on each side of the spine, by making a 10–12 mm incision through the upper layers of the skin and opening a small pocket between the layers of connective tissue where the cement disc was placed.

The wound was closed intracutaneously with a resorbable 4.0 suture (Polysorb, reference number SL-691, Tyco Healthcare, Gosport, UK). Immediately after operation, the rat was given 1.0 mL physiological saline solution subcutaneously, to avoid dehydration. During the first two postoperative days, 0.05 mg/kg buprenorphine (Temgesic, reference number 08 61 88, Sheringer Plough, Brussel, Belgium) was administered subcutaneously for analgesia. The rats were allowed to move freely in the cages directly after surgery. At the end points the implantation sites were macroscopically assessed for presence of tissue reactions. The implants were then collected by careful dissection, along with 5 mm of surrounding tissue, by first separating the dermis and hypodermis from the underlying muscle and bone and then excising a circular piece of tissue with the cement disc in the center.

#### 2.3.3. Enzymatic Digestion

The cement disc and epidermis layer were gently removed using scalpels, and the remaining subcutaneous tissue was cut into small pieces and enzymatically digested at 37°C for 90 min, in an enzyme mixture containing 0.2% hyaluronidase (hyaluronidase from bovine testes, reference number H3506, Sigma) in PBS and 0.5% collagenase (crude collagenase from* Clostridium histolyticum*, reference number C-6885, Sigma) in HBSS buffer (Hank's Balanced Salts, pH 7, Sigma). Digested tissue was filtered through 70 *μ*m cell strainer (BD Falcon) to separate the cells and remove debris, and the strainer was rinsed with PBS to keep as many cells as possible. The collected cells were spun down (720 g, 4°C, 6 min), the supernatant discarded, and the pellet was resuspended in MACS buffer (MiltenyiBiotec). The pellet was then spun down again (2200 g, 4°C, 6 min), the supernatant discarded, and the pellet resuspended again in MACS buffer and kept on ice until staining.

#### 2.3.4. Cell Staining and Flow Cytometry Analysis

Since acrylic bone cements are known as inert, permanent biomaterials a flow-cytometry-based method for evaluating only the surrounding tissue, and not the implant itself, was optimized from Ryhänen et al. [21]. The presence of immune cells was evaluated incubating the cell suspension, according to manufacturer's recommendation, with HIS36 antibody, specific for the macrophage marker ED2-like antigen, 0.2 mg/mL (BD Pharmingen); HIS48 antibody, specific for an antigen on granulocytes of rat origin, 0.5 mg/mL (BD Pharmingen); and APC/Cy7 anti-rat CD45 antibodies, specific for the leukocyte common antigen CD45 clone OX-1, 0.2 mg/mL (BioLegend) [22]. Afterwards, the cells were resuspended in PBS and strained through 40 *μ*m cell strainer (BD Falcon). The final solutions were read by BD LSR II flow cytometer (BD Bioscience). All data was processed by BD FACSDiva software (BD Bioscience) according to manufacturer's recommendations.

#### 2.3.5. Histological Analysis

The implant-tissue-complex was fixed in 4% phosphate buffered formaldehyde (reference number 02176, Histolab Products AB, Gothenburg, Sweden) at room temperature for 7–14 days. After fixation, the soft tissue at one end of the implant was cut and the cement disc was gently removed. The samples (soft tissue with implant removed) were mounted in paraffin, and several 7 *μ*m horizontal sections from each sample specimen were prepared on an automatic microtome (Microm HM 355 S, Thermo Scientific). Sections were mounted on glass microscope slides and stained with Mayer's hematoxylin and eosin-phloxine. Stained sections were scanned using a histology slide scanner (PathScan Enabler IV, Meyer Instruments, Houston, TX, USA) and evaluated for local histopathological response according to ISO standard 10993-6 [23].

#### 2.3.6. Radiopacity Evaluation

The radiopacity of the modified materials was evaluated and compared to the base material (OP) to determine if the material modifications had an influence on this property. The samples were scanned with a microtomography (*μ*CT) (SkyScan 1176, Bruker, Kontich, Belgium) both in vivo and ex vivo. For the implants analyzed in vivo, a source voltage of 90 kV, current of 278 *μ*A, exposure time of 90 ms, and a Cu filter of 0.1 mm were used. For the implants analyzed ex vivo, a source voltage of 80 kV, current of 313 *μ*A, exposure time of 1350 ms, and a Cu + Al filter were used. The images were reconstructed with NRecon software (Bruker) using a pixel size of 8.7 *μ*A, ring artifact correction of 7, smoothing of 2, and beam hardening correction of 30%.

### 2.4. Statistical Analysis

Statistical analysis was performed in IBM SPSS Statistics version 21 (IBM, Chicago, IL, USA) using a one-way ANOVA at a significance level of *α* = 0.05. Dunnett's (2 sided) post hoc test was used with OP as a control.

## 3. Results

### 3.1. In Vitro Study

**Figure 1:**
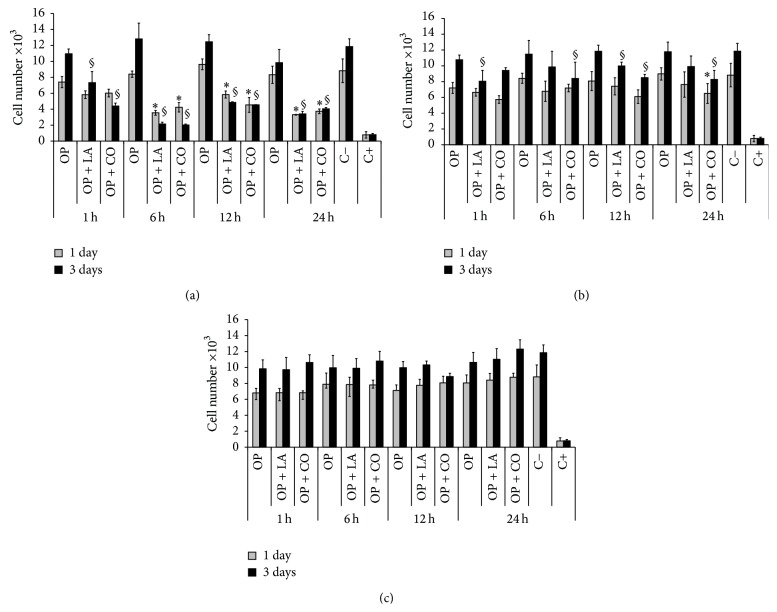
Viability of Saos-2 cultured for 1 and 3 days in 1, 6, 12, and 24 h extracts prepared with OP, OP + LA, and OP + CO. (a) Undiluted extracts; (b) 4-fold diluted extracts; (c) 10-fold diluted extracts. For each extract time, *∗* and § indicate statistical differences (*p* < 0.05) between each sample and OP at 1 day and 3 days, respectively. The error bars represent the standard deviation of the mean. Four replicates per sample were included in the assay. C− refers to negative control (fresh media) and C+ refers to positive control (0.1% triton).

Figure 1 shows the number of cells alive after incubation for 1 and 3 days with extracts as obtained (undiluted), diluted 4-fold and diluted 10-fold. Regarding undiluted extracts (Figure 1(a)), the cell number was not significantly different (*p* > 0.05) between OP-extracts at any time point. However, the number of cells alive after 1 and 3 days of incubation with additive-containing OP-extracts (OP + LA or OP + CO) was significantly lower in comparison to OP-extracts (*p* < 0.05), for most extract times. The extract time had an influence on the cell number, with higher cell number for 1 h extracts and lower number of cells alive for 6 h cement extract. Finally, an increase in cell number was observed from 1 day to 3 days for all OP-extracts. In contrast, cells did not grow in most of the OP + LA- and OP + CO-undiluted extracts prepared for 1, 6, and 12 h.

In diluted 4-fold extracts (Figure 1(b)) there were a similar number of cells in most of the compositions at 1 day (*p* > 0.05). At 3 days, the cell numbers in OP-extracts were statistically higher (*p* < 0.05) than OP + LA-extracts prepared for 1 and 12 h and than OP + CO-extracts prepared for 6, 12, and 24 h. Although the extract time did not show a clear trend on the number of cells alive, extracts of OP + LA and OP + CO prepared for 1 h had lower cell numbers after 1 and 3 days of incubation. Interestingly, cells showed a prominent increase after 3 days of incubation in all 4-fold diluted cement extracts.

When 10-fold diluted extracts were used (Figure 1(c)), no statistical differences (*p* > 0.05) were observed between any cement formulations. Moreover, the cell number in most of the cement extracts was similar (*p* > 0.05). No evident influence of the extraction time was observed and cells were able to grow after 3 days of incubation.

### 3.2. In Vivo Study

#### 3.2.1. Animal Model

All animals tolerated the surgery and the postoperative period well, and macroscopic assessment of the implant sites during the study period and at the end points showed no signs of tissue irritation or prolonged immune reactions, such as hematoma or edema.

#### 3.2.2. Flow Cytometry Analysis

**Figure 2:**
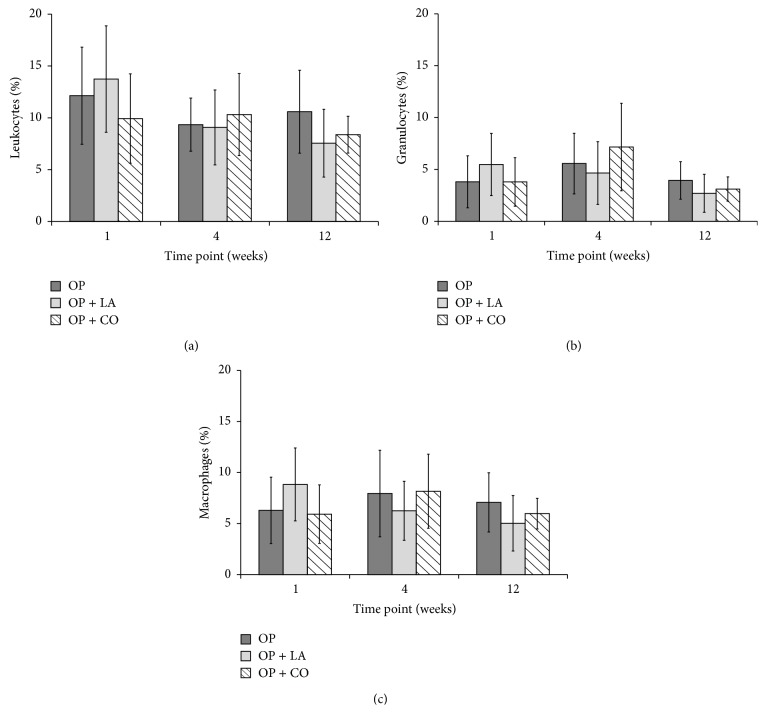
Evaluation by flow cytometry at 1, 4, and 12 weeks after implantation showed no statistical difference in the cell populations present in the tissue surrounding the modified material compared to the base materials. Immune cell populations are shown as mean percentage of entire cell population in each tissue sample. The error bars represent the standard deviations of the mean, with 6–10 replicates per group.

The presence of leukocytes and the leukocyte subpopulations macrophages and granulocytes around the implantation sites was evaluated by flow cytometry and is presented in Figure 2. No statistical differences (*p* > 0.05) were found between the populations of immune cells present in the tissue surrounding the different materials, indicating that there were no significant differences in the immune response to the modified PMMA cements (OP + LA and OP + CO) compared to the base cement (OP). No delayed immune response appeared to be triggered; there was no apparent increase in overall presence of immune cells over time.

#### 3.2.3. Histological Analysis

**Figure 3:**
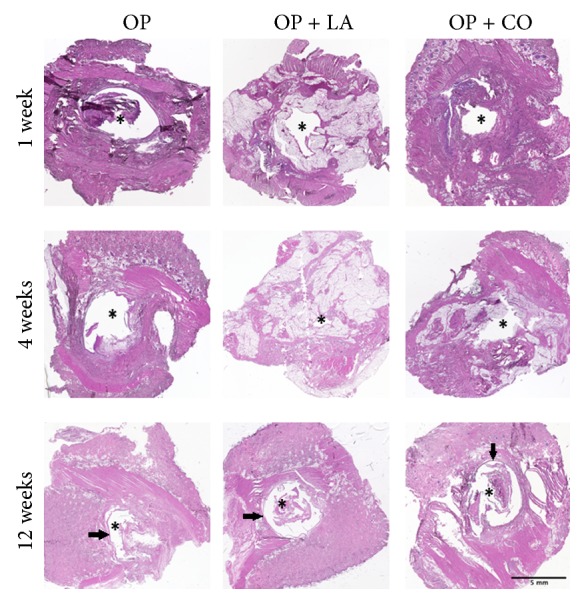
Representative histological sections of tissue explants from 1, 4, and 12 weeks, cut horizontally and stained with hematoxylin and eosin. The implant space within each section is marked by an asterisk. Arrows indicate fibrous capsule. Scale bar applies to all sections.

Assessment of histological sections stained with hematoxylin and eosin confirmed the macroscopic evaluation results. None of the material compositions caused any toxic reactions in the tissue surrounding the implantation sites. Also, no difference in tissue response between the base cement and the modified cements was visible, keeping in mind that the tissue surrounding the implants differs somewhat in composition (distribution of, e.g., fat and muscle tissue) between the implant locations, as the location in the body differs. Furthermore, for the assessed time points, no abnormal tissue organization could be seen at the implantation sites, apart from the necessary wound healing. At the later time points, the formation of a fibrous capsule had started around all implants. Representative histological sections are shown in Figure 3.

#### 3.2.4. *μ*CT Imaging

**Figure 4:**
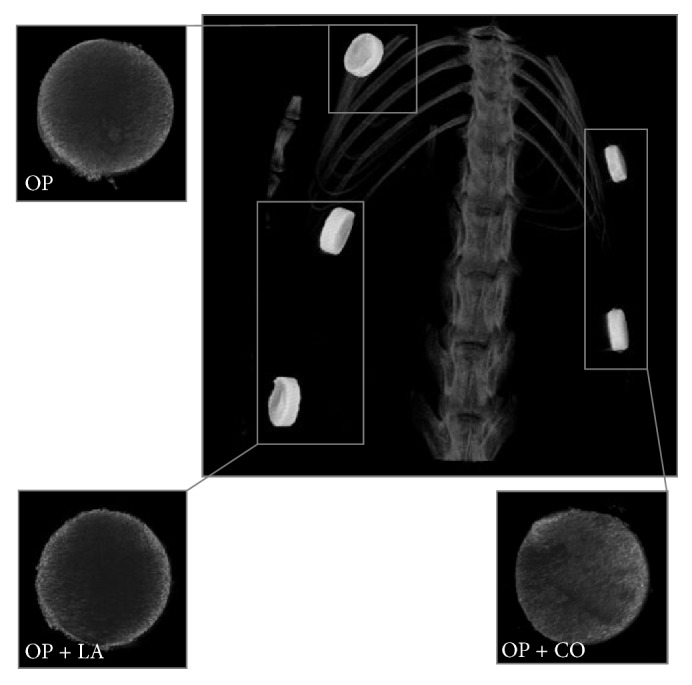
Representative scans of all material compositions in vivo (large picture) and ex vivo (small pictures). The modified materials have the same radiopacity as the base material and are equally visible in vivo. Five implants out of eight are observed in this image.

Radiopacity and in vivo visibility of the modified materials were evaluated by *μ*CT and found to be equal to the base material. Representative images are shown in Figure 4.

## 4. Discussion

We have previously shown that fatty acids and triglyceride oils are able to substantially improve the mechanical properties of acrylic bone cements in terms of lowering their elastic modulus [17]. However, the effect of their addition on surrounding cells and tissues has not yet been investigated. Therefore, the aim of this work was to bring light to the cytotoxicity and immune response to the materials through a combined in vitro and in vivo study. Since PMMA is commonly used in vertebroplasty, where it is in contact with bone, the cytotoxicity of the materials to osteoblast-like cells was tested. However, for simplicity and to minimize the invasiveness of the surgical procedure, a soft tissue site was used for the in vivo model. A future study will however evaluate the host response in a bony site, to more closely mimic the clinical situation.

In the in vitro study, cells were incubated in the presence of cement extract. The extracts were evaluated undiluted as well as diluted 4- and 10-fold to more closely simulate the in vivo conditions, in which physiological fluid flows through the porous structure of cancellous bone within the vertebra [24, 25]. The results showed that whereas undiluted extracts of OP were harmless, undiluted extracts prepared with additive-containing cement reduced the number of cells alive (Figure 1(a)). This could be associated with either the LA or CO itself or with a delayed reaction of PMMA cement in presence of these additives, causing a higher release of monomer into the extract, as discussed elsewhere [17]. The reduction in cells observed was similar regardless which additive, LA or CO, was added to the cement, even though the amount added (1.5 and 12.3 wt%, resp.) to the cement was very different. This indicates that LA has a larger effect than CO in terms of delaying the polymerization process. The higher cell numbers observed with undiluted extracts prepared for 1 h were associated with the short time in which the cement was in contact with the medium. In contrast, extracts prepared for 6 h showed lower cell numbers than those prepared for 12 h and 24 h, suggesting that most of the toxic species were released at earlier times. Interestingly, by diluting the cement extracts only 4-fold, the cell numbers after 1 and 3 days of incubation were similar to that of fresh media for most of the samples, and cells were able to grow during this period of time (Figure 1(b)). Therefore, as expected, while incubating the media with 10-fold diluted extracts, the cell number in most of the samples was not statistically different to that of fresh media (Figure 1(c)).

The cytotoxic potential of PMMA cements has been known for a long time [26]. This behavior has been associated with the polymerization reaction, which causes the release of heat as well as free radicals with high reactivity. Some of these radicals may escape from the cement area and react with biological molecules, thus causing cell damage [27]. In our in vitro studies, we simulated physiological processes and transport phenomena of body fluids that occur in vivo [24, 25] by performing dilutions of the cement extracts. Fourfold dilution of the extract allowed overcoming their cytotoxicity. Similarly, other in vitro cell studies on PMMA-based materials have used 2–16-fold diluted extracts (prepared following a 3 cm^2^/mL ratio or 0.1 g/mL), in some occasions only finding no difference to negative controls for 8-fold dilutions or above [28, 29]. Previous in vitro cytotoxicity studies on calcium phosphate based bone cements have also used similar dilutions [30]. While in vitro cytotoxicity studies may give indications on differences compared to standard materials, the in vivo response is important to evaluate in order to have a more accurate prognosis of the material behavior in the clinics. In this study, we used a minimally invasive subcutaneous screening using a rat model, following a similar procedure as the one used by Hulsart-Billström et al. [31]. Local tissue response can be considered one of the most important factors of biocompatibility. The biocompatibility of novel biomaterials is generally evaluated based on the in vivo inflammatory responses and the fibrosis formed around the implant. A mild inflammation is expected for all foreign materials, including commercial biomaterials, and is recognized as part of the body's foreign body response [20]. In subcutaneous screening models, early time points (in this case one and four weeks) are normally characterized by increased acute or chronic inflammation and limited granulation tissue and foreign body reaction. In contrast, at later time points (in this case 12 weeks), acute and chronic inflammation are absent, and granulation tissue and foreign body reaction have significantly decreased from their peak values, which is usually reached around three weeks [32]. Initially, during this local tissue response neutrophils are present in the highest numbers, but these cells are short-lived and remain at the implant site only for the length of their lifespan of a couple of days. At later time points, monocytes have migrated to the implant site and differentiated into mature macrophages, which are capable of staying at the site for long periods of time, up to several months in some tissues. They can also form foreign body giant cells that remain for the duration of the biomaterial's implantation [33, 34].

In this study, the response pattern observed was quite comparable to the earlier findings for subcutaneous models [33]. Histological evaluation also showed a tissue response and healing tissue organization comparable to PMMA-based materials implanted subcutaneously [35] and intramuscularly [36]. The local soft tissue was analyzed for presence of cell populations typical of inflammation—leukocytes, granulocytes, and macrophages—using flow cytometry. No delayed immune response appeared to be triggered as there was no change in overall presence of immune cells during the time of implantation. The grand majority of leukocytes present at the implant sites were macrophages and granulocytes, indicating that a normal inflammation reaction of the nonspecific immune response was taking place, rather than recruitment of the B- or T-lymphocytes of the specific immune response. Both modified cement compositions (PMMA supplemented either with linoleic acid or with castor oil) also showed a response profile completely comparable to that of the PMMA base material, which is already in commercial use. To the best of the authors' knowledge, flow cytometry has not previously been used to analyze the response of PMMA in vivo.

In summary, the low-modulus cements caused a decrease in in vitro cell viability in comparison to the nonmodified cement when using nondiluted cement extracts. However, for the worst-case, 6 h extracts, by diluting them only 4-fold, the cell growth showed no differences between samples and neither in comparison to the fresh media. In the in vivo study, the flow cytometry analysis and the histology results showed no significant differences between unmodified cement and the low-modulus cement samples. This is the first time low-modulus acrylic bone cements have been evaluated in an in vivo model. While these results are promising, the mechanical functionality of these types of cements remains to be evaluated in vivo.

## 5. Conclusions

In this study, we showed that two types of low-modulus PMMA-based bone cements have comparable in vitro cytocompatibility to commercially available conventional PMMA cement after only a small degree of extract dilution. Moreover, under in vivo conditions, all materials showed a similar biocompatibility and inflammatory response to conventional PMMA cement. The radiopacity of the cement also appeared unaffected by the modifications.

## Figures and Tables

**Table 1:** Design of the in vivo study. For each animal the end point as well as number and type of implants is specified. Total number of implants per formulation was 48, with 16 samples per end point. Six–ten samples were used for flow cytometry and the rest for histology.

Animal	End point (weeks)	Number of OP implants	Number of OP + LA implants	Number of OP + CO implants
1	1	4	2	2
2	4	2	2	4
3	12	2	4	2
4	1	4	2	2
5	12	2	2	4
6	4	2	4	2
7	12	4	2	2
8	12	2	2	4
9	1	2	4	2
10	4	4	2	2
11	4	2	2	4
12	1	2	4	2
13	4	4	2	2
14	1	2	2	4
15	4	2	4	2
16	12	4	2	2
17	1	2	2	4
18	12	2	4	2

**Total number**		48	48	48
